# An analysis of changes in wellbeing during the COVID-19 pandemic in the UK

**DOI:** 10.1007/s44155-022-00009-x

**Published:** 2022-04-25

**Authors:** Jen Murphy, Mark Elliot

**Affiliations:** School of Social Sciences, University of Manchester, Oxford Road, Manchester, M21 9DN Greater Manchester UK

**Keywords:** Well-being, COVID-19, Social gradient, Resilience

## Abstract

**Purpose:**

We investigated the trajectory of wellbeing over the course of the first wave and sought to determine whether the change in wellbeing is distributed equally across the population. Specifically we investigated pre-existing medical conditions, social isolation, financial stress and deprivation as a predictor for wellbeing and whether there were community level characteristics which protect against poorer wellbeing.

**Methods:**

Using online survey responses from the COVID-19 modules of Understanding society, we linked 8379 English cases across five waves of data collection to location based deprivation statistics. We used ordinary least squares regression to estimate the association between deprivation, pre-existing conditions and socio-demographic factors and the change in well-being scores over time, as measured by the GHQ-12 questionnaire.

**Results:**

A decline in wellbeing was observed at the beginning of the first lock down period at the beginning of March 2020. This was matched with a corresponding recovery between April and July as restrictions were gradually lifted. There was no association between the decline and deprivation, nor between deprivation and recovery. The strongest predictor of wellbeing during the lock down, was the baseline score, with the counterintuitive finding that for those will pre-existing poor wellbeing, the impact of pandemic restrictions on mental health were minimal, but for those who had previously felt well, the restrictions and the impact of the pandemic on well-being were much greater.

**Conclusions:**

These data show no evidence of a social gradient in well-being related to the pandemic. In fact, well-being was shown to be highly elastic in this period indicating a national level of resilience which cut across the usually observed health inequalities.

## Introduction

In March 2020, in response to the rising prevalence the disease in the UK, the government followed many others in declaring a ‘lock-down’ where citizens were required to stay at home beyond a very limited number of sanctioned reasons [1]. Mass gatherings were banned, travel was restricted. Leaving the home was restricted to those working in so-called ‘key-worker’ roles such as healthcare, education and the food system. Non key-workers were permitted to exercise alone outside of the home once daily, and to make trips for essential supplies. All non-essential services were closed including shops and leisure facilities. Non-emergency care was seriously compromised with many routine care services stopping for several months including cancer diagnostics, chemotherapy, surgery and outpatient clinics.

We hypothesise that as a result of the pandemic and the accompanying lockdown, wellbeing has been impacted and that there are likely to be widespread indirect effects important to policymakers and health professionals as the population recovers. Studies using the UK Household Longitudinal Study show that there has been a deterioration in the average mental health of respondents when comparing data waves before and early in the pandemic. Proto and Quintana-Domeque [2] report that the extent of the deterioration varies by ethnicity and by gender. Pierce et al. [3] explored the trend in UK mental health, demonstrating that there had been a deterioration compared with pre-COVID-19 trends, highlighting greater increases for younger adults, women, and people living with young children. Google Trends data showed an increase in search terms for boredom, loneliness, worry and sadness indicating mental health was impacted by the lockdown [4]. A study of 6142 adults from 18 countries within the Middle East and North Africa in May and June 2020 concluded that the pandemic was associated with a mild psychological impact, with half the sample reporting feelings of being horrified, helpless or apprehensive [5]. Holmes et al. call for resources to be deployed to understand the varied effects, collecting data and conducting multidisciplinary research to ensure efficient targeting of policy mitigations [6]. The authors specifically reference mental health, and the growing threat of virus mitigation measures, alongside the potential physiological effects of contracting the virus on brain function and mental health in patients testing positive with COVID-19.

In this paper, we use the UK Household Longitudinal Study, Understanding Society [7] to investigate the trajectory of wellbeing over the course of the first wave (defined as April–July 2020) and analyse whether any change in wellbeing is associated with social and demographic factors. In the next section we discuss the background to the pandemic and factors which may affect wellbeing. In Sect. 3, we introduce the dataset used and the methods applied. Results are presented in section 4. Section 5 contains the discussion of findings, followed by an appraisal of the strengths and weaknesses of the work in Sect. 6. We conclude in Sect. 7.

## Background: factors affecting wellbeing in a pandemic

The global pandemic has been a period of extreme stress and challenge. Communities and individuals have needed to rapidly adapt to the developing situation and in many instances, significant adversity. The ability to adapt to the changing health and social landscape at an individual and community level may affect wellbeing, and the extent to which communities and individuals are resilient to these stresses may influence the nature and duration of this impact.

Morton and Lurie [8] present domains of community resilience. At an individual level, physically and mentally well individuals are more resilient, with better underlying population health contributing to this individual resilience. Conversely, individuals with poorer underlying health, or with under-treated chronic conditions, find it more difficult to re-establish a health promoting way of life in the aftermath of adversity and are thus less resilient to any challenges they face. Individual mental resilience enables individuals to adopt positive adaptations in response to (and despite) external stress factors, but this mental resilience can be impaired by changes to the normal social life of an individual, for example through disruption of social networks. This type of disruption impacts all actors within the network and thus also leads to reduced population health at the community level.

The pandemic has disrupted our social existence and many of the support structures in place to support those with poor underlying mental health and other chronic conditions [9]. Outcomes for individuals may therefore have been impacted by not just by their own personal resilience, but also by the adaptation of their community and the resilience of the organisations upon which their communities depend.

Controlling the spread of COVID-19 continues to be a priority to protect the ability of the healthcare system to provide care for those who need it, and to reduce the number of excess deaths attributable to the disease. However, the measures taken have impacted incomes, social contact and job security. These factors are all known to contribute to an individual’s ability to live a healthy life and so we can expect to see an impact on long term health [10]. The Trussell Trust reported a rise of 122% in emergency food parcels for children during March 2020, compared to 2019 [11]. Those on a low wage, in particular the young, and women, were seven times more likely to work in sectors required to close by the COVID-19 restrictions with a third of employees in the bottom decile of the income distribution working in a closed sector, compared with only 5% of those in the top decile [12]. Economic contraction is expected to lead to an expected additional 3.5 million claims for universal credit from the UK welfare system [13]. As the Health Foundation have observed, pre-existing inequalities are likely to cause uneven impacts of the virus, and it follows that complex patterns of health inequity will result [14].

For many people in the UK, the pandemic restrictions have either reduced incomes, or increased the threat of financial stress in the future [15, 16]. This may continue for some time, a recession is underway with large scale unemployment [17]. Unemployment is associated with excess mortality [18]. Individuals need money to meet their material needs and to participate and engage in health promoting activities, or being able to afford fresh goods and the time to prepare meals using them. Having insufficient money is stressful, and living with disadvantage can make a person more likely to engage in unhealthy behaviours. A systematic literature review by Benzeval et al. [19] has shown that the effect that having insufficient financial resources has on health, can further impede individuals’ education and employment causing an ill-health and income negative feedback loop.

Age can be a factor in mental health. A study of older adults in Hong Kong showed that during the 2003 Severe Acute Respiratory (“SARS”) pandemic, suicides in the age 65+ age group increased by 30%. This increase was attributed to fears of being a “burden” to family during the outbreak, but also social disengagement, mental stress and anxiety [20].

For patients living with a long term condition, social engagement and access to informal healthcare services such as support groups is part of ongoing self management. For example, Reeves et al. [21] studied 300 patients with diabetes or chronic heart disease living in deprived areas of the North West of England. The authors found that self management, and physical and mental health were supported by social involvement with groups and people. Patients increased their use of their social networks as their care needs increased, showing a dynamic effect that was reflected in financial savings to the care providers. Social networks act for this cohort as a support to and a substitute for more formalised health care services.

This social network effect may have been significantly disrupted by the COVID-19 pandemic leading to increased social isolation and potentially loneliness. At a time when care services were stretched by patients requiring care for COVID-19, patients living with a long term care need may have needed to draw more on this social network for their own self-management as access to formalised healthcare settings became restricted [22]. Access to groups, socialising and networks including family and friends was at the same time restricted for all, and particularly for those living with significant co-morbidities who were instructed to “shield” for a period of 3 months [23]. Support for self-management of long term conditions is a networked and collaborative construct, as opposed to merely based on the action of individuals, and so a time of significant social isolation may well have caused a break down in self-management of health and wellbeing for those with longer term care needs [24–26]. Not everyone who experiences social isolation, feels lonely and indeed loneliness may occur without social isolation, however Emerson et al. [27] demonstrated that loneliness was also associated with wellbeing for a representative sample of people with and without a disability. Coyle and Dugan [28] studied older adults, showing that loneliness is associated with poorer mental health.

The highly infectious nature of the COVID-19 virus necessitated significant organisational changes for health care services on a global scale. In a multinational survey, resource reallocation from chronic disease to COVID-19 disrupted the continuity and the quality of care across all countries, with specific impact on diabetes, chronic obstructive pulmonary disease and hypertension [29]. Elective surgeries and outpatient clinics were cancelled with many care appointments postponed and most care moving to remote provision by teleconsulting [30]. In the early stages of the first wave, evidence of risk factors for infection and mortality had not yet emerged and a crisis in demand for respiratory care de-prioritised other areas of the health care system. Health care service overcrowding affected the resourcing and facilitation of ongoing treatment and palliative care for conditions such as cancer [31]. Diagnoses were reported to be delayed as services for screening and testing were suspended and many patients were reticent to engage with healthcare services for fear of contracting the virus in a hospital or other setting [32]. Maringe et al. [33] predict over 3000 excess cancer deaths in the next five years as a result of delays to diagnostic and treatment services, in a sample of 93,607 patients suffering from one of four specific tumour types. In the case of diabetes care, the strain on emergency health care services required many medical staff to be seconded to alternative roles, further compromising the availability of specialist services. Nagi et al. [34] report a reduction in acute admissions for diabetes and related endocrine disorders and a reduction in investigations. Standard outpatient clinics were closed and cancellation of face to face clinics alongside a reduction in availability of services, caused the care to be delivered to be sub-standard in addition to there being concerns of “important unmet clinical need”.

Using questionnaire responses from the COVID-19 modules of Understanding Society [7], we examine the change in wellbeing for a sample of respondents in England^1^ during the lock down period associated with the UK’s ’first wave’ of COVID-19 infections using the twelve question General Health Questionnaire as a proxy measure for wellbeing.

We ask the following research questions:

1. Is the reported initial decline in wellbeing distributed equally across all groups regardless of deprivation?
2. Is the reported initial decline in wellbeing the same for those with pre-existing medical conditions?
3. Did wellbeing change overall during the course of the first wave?
4. Has any overall change in wellbeing been experienced equally across those in deprived areas or with pre-existing medical conditions?
5. Are there community level characteristics which are protective against poorer wellbeing?

## Data and methods

The data are taken from the first four waves of the Understanding Society COVID-19 survey, with wave nine data used as a baseline [7]. The outcome variable is the General Health Questionnaire (GHQ-12) caseness score for each survey.

The twelve item General Health Questionnaire (GHQ) is a validated measure of mental distress and is considered robust in longitudinal data samples [35]. Each question is rated on a 1–4 Likert scale with the answer 4 indicating the response associated with the poorest wellbeing for each question. The measure includes generalised questions about concentration, sleep, decision making, feelings of worthlessness, confidence, stress, and happiness. The GHQ index variable is the sum of responses to the twelve questions. The maximum score for a complete questionnaire is 48 and the minimum is 12. Question and response texts are given in Appendix A.

The caseness variable is computed by recoding all responses to a binary 1/0 value. Responses of 1 and 2 are recoded to 0, indicating no change from usual, whereas 3 and 4 and recoded to 1. Summing over the twelve questions gives the caseness score. A caseness score of 1–2 is considered to indicate mild psychiatric illness [36].^2^

Valid cases are selected as those who responded to all five waves of data.^3^

The longitudinal response rate to waves 1 through 4 of the COVID-19 survey is 21.8%, representing 9,603 valid cases UK wide. 58.3% of respondents were female, compared with 53.2% in the survey sample. Of these 8379 resided in an English Lower Super Output Area during the wave 1 COVID-19 data collection and were included in the analysis.

The mean age of respondents in England is 55.4 years (SD 15.6) compared with the overall Understanding Society sample mean age of 49.1 years (SD 19.3)

Logistic modelling of longitudinal response across the four waves of data collection, identified sex, ethnicity, age and baseline GHQ-caseness (as recorded in wave 9 of the main survey) as predictors of response.

There are only 786 valid English non-white respondents (9.4%). In the overall sample 20.3% are non-white ethnicities suggesting an non-random missingness and an under representation of BIPOC communities within the data. This is accounted for in the models by including ethnicity as a co-variate regardless of its effect.

The extent of decline in wellbeing is measured by the change in GHQ caseness score between wave 9 of the main survey and wave 1 of the COVID-19 survey. The progression of wellbeing during the first pandemic wave is defined as the change in this score between waves 1 and 4 of the COVID-19 survey. The base line is selected as wave 9 of the main survey as this was the most recent dataset available at the time, and data collection occurred well before the emergence of the pandemic.

The response to a question about loneliness is used in different forms in both models. Modelling the decline, we compared the loneliness response for wave 1 of the COVID-19 data collection with the baseline and constructed a categorical variable to capture the trajectory of loneliness for respondents with four responses:

- remaining lonely (having been lonely previously),
- becoming lonely (having not felt lonely before),
- no longer feeling lonely (having felt lonely before),
- not having experienced loneliness either before or at the beginning of the lock-down.

Using this variable as a series of dummies within the model provided the same result as simply using the response to the first wave of COVID-19 data collection and therefore—for reasons of parsimony—we use the variable *lonely* in the model. The recovery model uses a cumulative score to capture persistent or frequent loneliness over time.

The variable *income_decrease* is derived from the household composition and household earnings amounts. The household makeup was summed using variables^4^ which correspond to the number of adults residing in the household and those^5^ which correspond to the number of children within the household. This was then equivalised to account for the differing costs of adult and child residents using the formula given by the OECD as an appropriate method for equivalence [37]: $household\_equivalence = 1 + 0.5(adults) + 0.3(children)$.

The income change was computed using the baseline and third wave household income values, equivalised to an annual figure^6^

The economic impact of the income decrease was calculated by scaling the change in income between the baseline and wave 3, by the equivalised household size and then recoding this variable to show whether or not income had decreased during the period of the lockdown.

Using respondent geography, each response was assigned a deprivation decile corresponding to home location for wave 1 of the COVID-19 data collection using the 2019 updated English Indices of Multiple Deprivation (IMD) [38]. The variable *community_cohesion* was compiled from four questions asked in the third wave of data collection, in June 2020. Questions asked whether a respondent spoke regularly with neighbours, trusted those who live in their community, found neighbours to be helpful and whether or not they got along with people in their community. The responses on a Likert scale, were re-coded and summed to give a composite score for the respondent’s neighbourhood.^7^

**Fig. 1:**
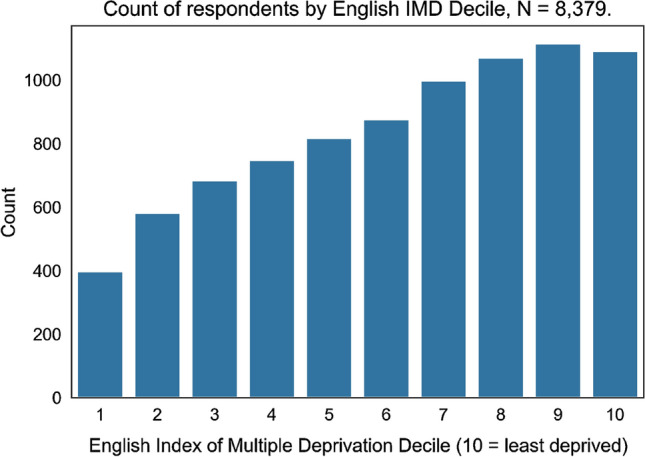
Distribution of respondents by IMD Decile

In 129 cases Lower Super Output Area (LSOA) changed during the period. 5 cases changed more than once, of which 4 reflected a move away and then back to an LSOA of origin. LSOA of origin is defined as the relevant LSOA for determining deprivation. Respondents are skewed towards areas of lower deprivation. 2206 respondents live within LSOA’s ranked in the bottom two deciles for deprivation, compared with 5150 in the top two deciles. The mean IMD decile for a respondent was 6.3 (SD 2.7) where 10 reflects the least deprived areas (Fig. 1).

43% of the men (N = 1523) and 47% of the women (N = 2.287) in the analysis had no underlying health conditions. Of those reporting an underlying health condition, 57% were women (N = 2602) in line with the overall sex distribution of respondents.

Parameters in the regression model were estimated using using ordinary least squares regression with wave 4 longitudinal weights, using $R^2$ as a measure of model fit. The variables used in the analysis are described in Table 1.^8^

**Table 1:** Variables in the analysis

Variable	Mean	Std	Missing	Notes
	(male/female)	(male/female)		
wellbeing_decline	0.606/1.30	2.87/3.67	0	Change in GHQ-12 caseness score from wave 9 of the main survey (*i_scghq2_dv*) to wave 1 of the COVID-19 survey *ca_scghq2_dv*).
baseline_wellbeing	1.24/1.80	2.58/3.04	0	GHQ-12 caseness score from wave 9 of the main survey (*i_scghq2_dv*).
wellbeing_recovery	0.458/1.00	2.54/3.27	0	Change in GHQ-12 caseness score from wave 1 (*ca_scghq2_dv*) to wave 4 (*ca_scghq2_dv*) of the COVID-19 survey. Positive values indicate a reduction in the score, an improvement in wellbeing.
age	56.8/53.3	15.4/15.7	0	Age in years, calculated from birth year (renamed from survey variable: *ca_age*).
sex			0	Sex, binary. 1 = Female, 0 = Male. (recoded from survey variable: *sex_dv*).
community_cohesion	14.8/14.9	2.7/2.8	20	Variable (derived from questions on neighbourhoods: *scopngbhh, nbrcoh3, nbrcoh2 and nbrcoh4*).
imd_decile_2019	6.3/6.2	2.7/2.7	0	English Indices of Multiple Deprivation decile for the respondent’s LSOA as at wave 1 data collection [imputed from 38].
eth_minority			26	Binary variable to indicate white and non white ethnicities. 0 = White 1 = Black, Asian and Minority Ethnic (derived from the survey variable *race_dv*).
health_condition			0	Binary variable to indicate underlying health conditions. 0 = no health condition, 1 = health condition. Taken at July data collection as those diagnosed during the COVID-19 survey period are likely to have been living with symptoms and accessing care for an undiagnosed condition during the period. (derivation of the survey variable: *cd_ff_hcondhas*).
lonely			0	Binary variable to indicate experience of loneliness in the 4 weeks prior to the wave 1 data collection. 0 = has not experienced loneliness, 1 = experienced loneliness some times or often. (derived from the survey variable *ca_sc_lonely_cv*).
always_lonely	0.97/1.54	1.4/1.6	2	Sum of binary variables over waves 1 to 4 to give a score for persistent loneliness. Max = 4, min = 0. (calculated from derivations of four survey variables: *ca_sc_lonely_cv, cb_sc_lonely_cv, cc_sc_lonely_cv and cd_sc_lonely_cv*).
financial_crisis			3	Binary variable to indicate acute financial crisis at Wave 4. 1 = has accessed a food bank in the prior 4 weeks, 0 = has not (derived from the survey variable *cd_foodbank_cv*).
income_decrease			0	Binary variable to indicate worsening financial situation. 1 = house hold equivalised income has reduced from wave 1 to wave 4, 0 = income is the same or greater (calculated from multiple variables, see text).

The model was constructed in three steps (A, B and C) corresponding to underlying demographic factors (A), baseline GHQ scores (B) and variables which correspond to specific pandemic lock down phenomena (C). Non-significant variables are retained throughout as controls for model equivalence.

We note that using the same GHQ-12 questions from the Understanding Society survey and its predecessor the British Household Panel Survey from 1999 to 2016, Brown et al. [39] showed that under reporting bias for mental health was greater for men. For this reason we elected to produce separate models for men and women.

Initial model specifications are can be seen in Eqs. (1) and (2) below.

1 $$\begin{aligned}{\textit{wellbeing\_decline}} =& \beta _0 + \beta _1({\textit{baseline\_wellbeing}})\\& \,\,+\beta _2({\textit{lonely}}) + \beta _3({\textit{age}})\\&\,\,+\beta _4({\textit{community\_cohesion}}) + \beta _5({\textit{imd\_decile\_2019}})\\&\,\,+\beta _6({\textit{health\_condition}}) + \beta _7({\textit{eth\_minority}})\\ \end{aligned}$$

2 $$\begin{aligned}{\textit{wellbeing\_recovery}} =& \beta _0 + \beta _1({\textit{baseline\_wellbeing}})\\&\,\,+\beta _2({\textit{wellbeing\_decline}}) + \beta _3({\textit{age}})\\&\,\,+\beta _4({\textit{community\_cohesion}}) + \beta _5({\textit{imd\_decile\_2019}})\\&\,\,+\beta _6(\text {eth\_minority}) + \beta _7({\textit{health\_condition}})\\&\,\,+\beta _8({\textit{always\_lonely}}) + \beta _9({\textit{financial\_crisis}})\\&\,\,+\beta _{10}({\textit{income\_decrease}})\\ \end{aligned}$$

Missing data within the valid cases were imputed with the mean value for the variable, with the exception of ethnicity where “white” was imputed. The level of missingness within the selected cases is very low (see Table 1). However, as a precaution, sensitivity analysis was conducted by re-running the regression models after dropping all cases with missing values, and comparing to the models with missing values imputed. The models were stable with minimal change in the magnitude or direction of estimated coefficients.

## Results

**Fig. 2:**
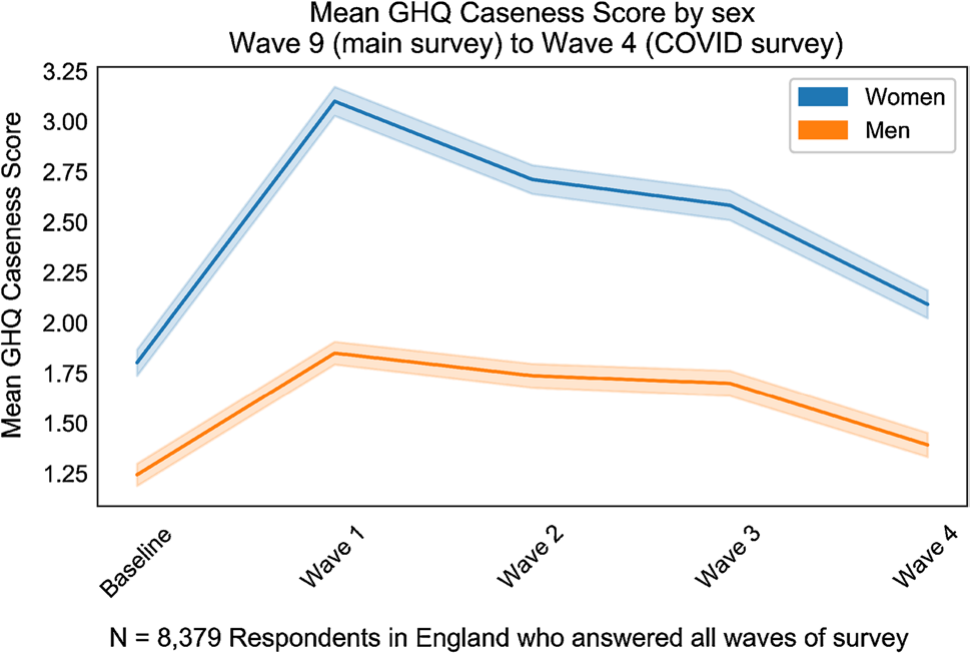
Mean GHQ Caseness Score over time, by Sex, with 95% confidence interval

**Fig. 3:**
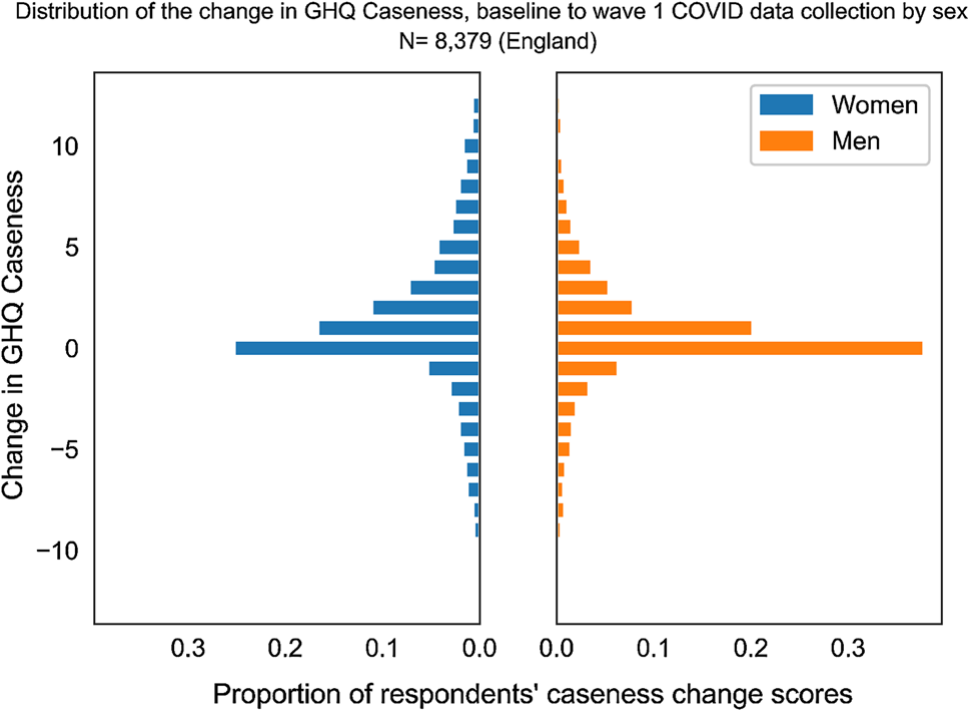
Distribution of the change in GHQ Caseness from baseline to wave 1, by sex

Mean wellbeing scores show a clear peak and decline with the peak occuring in April during the first wave of data collection. Mean scores have recovered to almost the baseline (main wave 9) by July (COVID-19 wave 4) when an end to shielding was announced and much of the economy reopened, albeit with restrictions in place to ensure continued social distancing. The increase in scores between the baseline and July 2020 are consistent with the trend in scores over time reported elsewhere (see Figs. 2, 3; Table 2) [3].

The difference between men and women in the mean figures for the baseline is statistically significant ($t=8.8$, $p<0.05$) as is the difference in the mean change for men and women between the baseline and April data collection ($t=9.3$, $p<0.05$).

Women suffered, on average an increase of 1.3 in the GHQ caseness score (72% increase on baseline) between wave 9 and the first COVID-19 wave, compared with 0.61 for men (51% increase on baseline). This indicates a difference in the impact of the pandemic on women’s wellbeing consistent with reports that women have been unequally impacted [40, 41].

**Table 2:** Mean caseness scores for males and females

	Mean	Std	Sex	95% CI	95% CI
				Lower	Higher
Baseline	1.80	3.05	Female	1.74	1.87
	1.24	2.58	Male	1.19	1.30
Wave 1	3.10	3.37	Female	3.03	3.17
	1.85	2.65	Male	1.79	1.91
Wave 2	2.71	3.34	Female	2.64	2.78
	1.74	2.78	Male	1.68	1.80
Wave 3	2.58	3.46	Female	2.51	2.66
	1.70	2.93	Male	1.64	1.76
Wave 4	2.09	3.24	Female	2.02	2.16
	1.39	2.79	Male	1.33	1.45

### Modelling the decline in wellbeing

An increase in the GHQ caseness score equates to a decline in wellbeing. Model 1C explains 36.7% of the variance in the wellbeing decline for women, and 33.1% for men (Table 3). Variance inflation factors were below two for all variables and all permutations of the model, indicating no problematic multi-colinearity. Pearson’s correlations for all variable pairs showed no correlations over 0.5.

The model build using a stepped approach shows that underlying demographic factors age, ethnicity, deprivation and pre-existing health conditions on their own (model 1A) explained little of the variance in GHQ scores ($R^2$ of less than 1% for both male and female models).

Introducing baseline scores from wave 9 in model 1B explained 25.9% and 26.9% of the variance for males and females respectively. Those with higher baseline scores, experienced less of a decline in wellbeing. Clogg test statistics (critical value 1.96) comparing parameter estimates between the two sexes show that the effect of this was stronger for women than for men. The difference in effect size however, although statistically significant, is small and does not offset the underlying result that women were affected more heavily than men (represented in these models by the large differences in the intercepts).

Model 1C shows the impact of introducing pandemic phenomena of loneliness and community cohesion into the estimates for well being. In this final model, for women, age, pre-existing health conditions, baseline and loneliness are associated with a decline in well being however age and underlying health conditions explain very little of the variance and have small effect sizes. The loneliness variable explains an additional 9.8% of variance (total $R^2$ of 36.7%) with a much larger effect size. For men the baseline and loneliness variables are significant. Introducing the loneliness variable explains an additional 7.2% of the variance (total $R^2$ of 33.1%). The difference in parameter estimates for the association between loneliness and the response variable between the sexes was not statistically significant (using a Clogg test).

Ethnicity and deprivation were not significant in the models for decline.

**Table 3:** Model 1: parameter estimates for the decline

		Male	Std. error	Female	Std. error
Model 1A	Intercept	0.316	(0.175)	1.393*	(0.141)
	age	0.005	(0.003)	− 0.009*	(0.002)#
	eth_minority	0.204	(0.188)	− 0.425*	(0.151)#
	imd_decile_2019	0.007	(0.017)	0.017	(0.014)
	health_condition	− 0.178	(0.104)	− 0.104	(0.083)
	$R^2$	0.2%		0.4%*	
Model 1B	Intercept	1.973*	(0.157)	3.383*	(0.126)#
	age	− 0.013*	(0.003)	− 0.029*	(0.002)#
	eth_minority	0.311	(0.161)	− 0.116	(0.129)#
	imd_decile_2019	− 0.021	(0.015)	− 0.021	(0.012)
	health_condition	0.291*	(0.090)	0.491*	(0.072)
	baseline_wellbeing	− 0.531*	(0.014)	− 0.595*	(0.011)#
	$R^2$	25.9%*		26.9%*	
Model 1C	Intercept	1.261*	(0.230)	1.834*	(0.181)
	age	− 0.001	(0.003)	− 0.01*	(0.002)#
	eth_minority	0.266	(0.154)	0.041	(0.121)
	imd_decile_2019	− 0.006	(0.014)	0.005	(0.011)
	health_condition	0.156	(0.086)	0.27*	(0.067)
	baseline_wellbeing	− 0.6*	(0.014)	− 0.671*	(0.010)#
	ca_lone	1.817*	(0.091)	2.518*	(0.067)
	community_cohesion	− 0.025	(0.015)	− 0.019	(0.011)#
	$R^2$	33.1%*		36.7%*	

*Indicates significant at the p<0.05 level. # indicates Clogg test statistic > critical value of 1.96

### Modelling the bounce back

The model for the recovery included further variables reflecting ongoing loneliness, and the onset of any acute financial crisis, as well as a measure of income stability. Women recovered a mean score of 1.0 (std 3.3), men to a mean of 0.46 (std 2.5). Compared with the baseline figure, the mean score had increased by 0.23 (std 3.3) but there was no statistically significant difference between men and women in this increase, at the 95% confidence level. The coding for the response variable within this model is reversed for more simple interpretation—a positive value relates to a decrease in the GHQ caseness score.

As for the decline, model A (Model 2A, Table 4) includes only variables related to sociodemographic characteristics. These models again described less than 1% of variance for males and females respectively.

The baseline figures introduced in model 2B increased the $R^2$ value to 19.4% and 27% for males and females, replicating the effect seen in the decline models. A higher baseline score from before the pandemic and a greater decline in the intial phase, were both predictive of a stronger recovery. Again their was a difference in the baseline parameter between the male and female models within women recovering more strongly for a given baseline score for both the pre-pandemic and the decline variables.

Model 2C introduces cohesion and loneliness as in the model for decline, but also includes variables which reflect the financial pressure of the lockdowns. The introduction of these pandemic factors explains a further 4.1% and 3.7% of variance for men and women respectively. Acute financial crisis was associated with a reduction in recovery for both males and females. Substantial changes in income over the period was associated with poorer recovery for women, but not for men. Although age and ethnicity were statistically significant in this model for females, the effect size is very small. Living in a cohesive community was significant for both sexes (and these parameter estimates were not statistically different between the male and female models).

The models indicate that the most important factor in the size of a person’s ‘bounce back’ is in fact the size of the original decline. Loneliness and acute financial crisis were statistically significant for both men and women, age, ethnicity and reduced income was significant for women but not men.

**Table 4:** Model 2: parameter estimates for the wellbeing recovery

		Male	(std err)	Female	(std err)
Model 2A	Intercept	0.148	(0.163)	1.195*	(0.126)
	age	0.003	(0.003)	− 0.01*	(0.002)#
	eth_minority	− 0.056	(0.175)	− 0.375*	(0.135)
	imd_decile_2019	0.029	(0.016)	0.014	(0.012)
	health_condition	− 0.122	(0.097)	0.052	(0.074)
	$R^2$	0.2%		0.3%*	
Model 2B	Intercept	− 0.805*	(0.156)	− 0.702*	(0.117)
	age	0.009*	(0.003)	0.007*	(0.002)
	eth_minority	− 0.205	(0.157)	− 0.324*	(0.115)
	imd_decile_2019	0.039*	(0.014)	0.026*	(0.011)
	health_condition	− 0.265*	(0.088)	− 0.235*	(0.064)
	baseline_wellbeing	0.257*	(0.016)	0.343*	(0.011)#
	wellbeing_decline	0.475*	(0.016)	0.538*	(0.010)#
	$R^2$	19.4%*		27%*	
Model 2C	Intercept	− 0.509*	(0.236)	− 0.423*	(0.176)
	age	− 0.001	(0.003)	− 0.005*	(0.002)
	eth_minority	− 0.127	(0.155)	− 0.316*	(0.115)
	imd_decile_2019	0.02	(0.014)	0	(0.011)
	health_condition	− 0.15	(0.086)	− 0.114	(0.063)
	baseline_wellbeing	0.375*	(0.018)	0.46*	(0.013)#
	wellbeing_decline	0.56*	(0.016)	0.628*	(0.011)#
	community_cohesion	0.039*	(0.015)	0.053*	(0.011)
	always_lonely	− 0.397*	(0.029)	− 0.403*	(0.021)
	financial_crisis	− 0.925*	(0.479)	− 0.965*	(0.244)
	income_decrease	− 0.039	(0.116)	− 0.351*	(0.088)
	$R^2$	23.5%*		30.7%*	

*Indicates significant at the p<0.05 level. #indicates Clogg test statistic > critical value of 1.96

## Discussion

Times of adversity and extreme stressful events have been linked with increased risk for poor well-being [42, 43]. There has been much concern in the media about the mental health and wellbeing impact of the COVID-19 crisis on people who have undergone an unprecedented change and restriction to their lives. Our research here suggests that although the first wave was associated with an overall decline in wellbeing, the removal of lock down restrictions was also associated with a recovery.

The study set out to uncover differences in the extent of the decline in wellbeing and subsequent recovery. We predicted that there would be an association of deprivation and decline in wellbeing. The removal of services and support networks for those with long term health conditions was expected to cause a greater decline in wellbeing and a reduced ability to recover. We also considered whether there would be effects attributable to ethnicity and sex.

We expected wellbeing trajectories to differ along sex, ethnicity, deprivation and underlying health lines, and that some of these differences would be explained by the impact of stress (specifically financial crisis), the level of community support experienced (community cohesion) and the extent of social isolation experienced by respondents (measured by loneliness).

The models here do not show the expected differences between groups. For this sample, wellbeing declined, but then gradually improved over the course of the first wave, returning nearly to the baseline level by July. Wellbeing in this context can therefore be considered to be elastic, that is to say that although there appear to have been negative impacts on wellbeing at the beginning of the first lock down, these impacts were lessened over time as restrictions reduced demonstrating a bounce back effect and a capacity for rapid recovery.

This is consistent with a level of adaptation, and may indicate some implementation of specific coping strategies by the respondents within the data. Indeed, some participants may have experienced a degree of post traumatic growth [43–45] whereby the imposition of adversity results in an improvement in wellbeing as those faced with the stress, draw on strengthened personal and social systems to thrive despite the situation in which they find themselves.

Deprivation appeared to show no impact on changes in wellbeing. The sample was skewed towards the less deprived deciles and so this may be a non-response issue, with those suffering the greatest deprivation, least able to engage with the survey, through poor mental health, or simply through having the means to respond online. Deprivation was assigned to respondents at the LSOA level which is in itself problematic as each LSOA represents approximately 1,500 individuals and thus may be heterogeneous with respect of deprivation.

Deprivation can be considered to be a pre-existing vulnerability which increases a person’s susceptibility to a disruption of any form and certainly deprivation could well be expected to have increased vulnerabilities to the social, economic and health impacts of the pandemic, particularly considering the evidence linking increased deprivation with poorer health outcomes [46]. However, the expected negative association of deprivation and health is not one that has been found universally in previous studies (e.g. [47–49]). The results of studies into health outcomes in deprived areas can sometimes be counter-intuitive and several investigations have shown that communities record better health outcomes than might be predicted from socio-demographic factors. These communities can be said to display ‘health resilience’ that is to say, they outperform expectations on certain measures.

No effect was detected here so it is possible that the expected social gradient in health has been cancelled out by additional resilience in the poorest communities. The social gradient implies that wealthier areas would experience less of a decline in well being but in fact many people in these communities were subjected to a level of stress to which they would be unaccustomed given their usual level of financial comfort and position of privilege in society. The threat of furlough, home working, home schooling and removal of a normal social life, may have been a sufficiently adverse effect on the better off that the mental health impact of the crisis has in fact been felt more equally than is usually the case for many other health measures. This observation may align with Holmes and Rahe [50] work on stress which proposes that life changes are the primary driver for reductions of wellbeing.

Women experienced a greater drop in wellbeing (a rise in the caseness score) than men, however at the end of the first wave there was no difference in the change in wellbeing between men and women. Self reported health is a combination of underlying health and reporting behaviour. Self reported mental health metrics are affected by misreporting, a potential impact of the continued stigma around mental health. Studying the same GHQ-12 questions from the Understanding Society survey and its predecessor the British Household Panel Survey from 1999 to 2016, Brown et al. [39] showed that this under reporting bias was greater for men. This may mean that the signal in these data showing a worse decline for women, is actually a factor of reporting bias. The baseline of the scores showed lower mental wellbeing for women than for men, the change was then greater for women than men, matched with a greater improvement. The uniformity of the elasticity across the sexes and the lack of lasting difference would tend to support a theory that the difference seen here can be attributed to reporting.

There was a low response rate amongst ethnic minority members of the panel with only half the expected number of respondents coming from an ethnic minority background of any type (approximately 10% from ethnic minorities in this sample, compared with 20% in the underlying survey panel). This necessitated the collapse of the detailed ethnicity variable to a binary ethnic minority/white measure. This is problematic because clearly people from different ethnic minority backgrounds are not homogeneous and may well have had experiences of the pandemic which varied by ethnicity for example due to the differing experiences of and relationships to family and community within different ethnic groups. The use of a binary variable also necessitates the categorisation of people with dual heritage into either “minority” or “white” and confounds British ethnic minority respondents with immigrant populations. Use of binary variables for ethnicity is problematic where the dominant research narrative considers the white perspective as central, and the ethnic minority perspective as “other”. Ethnicity was significant in the recovery model for women (model 2), An ethnic minority background was associated with a smaller “bounce back”. This may be because ethnic minority females suffered a smaller decline in wellbeing which was not detected in the modelling, or there maybe a resilience factors at play. However, the effect size and the overall contribution towards the explanation of variance small.

Age was significant for women in the decline of wellbeing and the recover but in both instances with a small effect size. Older people suffered a smaller decline in their wellbeing, and then a smaller recovery as the wave progressed. The mean age of respondents was skewed towards the older members of the panel. This may have reflected older people having more time on their hands relieved of their normal social lives and also perhaps the care burden for grandchildren, whereas younger adults were more likely to be juggling full time work from home whilst also caring for and schooling children. Poorer wellbeing in younger groups may also have contributed to non response.

Experiencing loneliness was predictive of a decrease in wellbeing in April for men and women and was a main contributor to the variance explanation in the model for wellbeing decline (Model 1C). Ongoing loneliness was statistically significant in the model for wellbeing recovery (Model 2C). Men and Women displayed the same effect. People who experienced continued loneliness using this measure, recovered less well as the pandemic progressed. There may be a stigma related to admitting that you are experiencing loneliness and so a bias in the response variable. The difference emerging between those who are lonely and those who are not is also indicative of the different ways in which people experienced the progressive loosening of restrictions. Some people opted to remain isolated, out of concern for their health, or because of shielding advice, whilst others made the most of new “freedoms”. Many of the coping strategies and adaptation mechanism which mediate resilience to external stress are constituted through family mechanisms and social interactions and relationships. Green et al theorise that “Multilevel attachments” are protective against life’s stresses [51]. For example, as Walsh et al observe families can adapt in times of crisis, and family relationships can mitigate against poor wellbeing [52]. Strength of the family and other social relationships can also therefore provide a path to adjusting to stressful situations and recovering from poor wellbeing. Similarly, Walsh et al write about the impact of belief systems and spirituality as a mediator for resilience [52]. During the pandemic, places of worship were closed and families were not able to be together. This measure of loneliness may well be reflecting this element of the restrictions and thus the framework of family resilience was disrupted by the pandemic and resulted in a reduction of wellbeing for those impacted through the removal of these important mechanisms.

Health inequalities follow a social gradient but in our final models for wellbeing decline (1C) and recovery (2C) deprivation was not associated with the response variable. The social gradient for health was not therefore replicated in these data when considering mental well being. This may be due to an overriding community effect which was present at the national level during the first wave. Many impacted directly by COVID-19 as a disease have suffered a devastating impact, through loss of their own physical health or bereavement. Indirect effects of the pandemic will take some time to uncover, but will include long term unemployment, and projected adverse outcomes in other health conditions as discussed in the introduction. These are likely to follow a social gradient but as the data used here were collected during the earliest stage of the crisis, the longer term impact of the pandemic’s duration and severity will not have impacted upon the mood of those responding.

The financial impact of lockdown differed widely dependent on employment sector and to an extent caring responsibilities as school age children remained for the most part, in the home. For respondents in the sample, an acute financial crisis resulting in food bank use was predictive of a worse mental recovery and this is consistent with expectations around stress and mental health. However, for women, a negative change in income also predicted worse recovery. That this is different for men and women is of interest. The income variable is set at the household level so this may reflect a response which differs by gender to the same phenomenon. It may also reflect the unequal caring burden placed upon women and in fact be a example of increasing marginal returns. Women within the analysis were already suffering worse well being and a greater decline in the pandemic. The addition of reduced income may have thus been incrementally more stressful for them, given that they were, already suffering poorer wellbeing.

We expected that in communities where people are more likely to speak to each other and where respondents report having neighbours they can rely on for help, the negative impact of the pandemic would be mitigated. In the model for decline, the measure of community cohesion was not significant however in the recovery, this variable was associated with a stronger recovery for both men and women. This perhaps may be indicative of the physical reality of the lockdown, during the lockdown phase access to the social capital that community cohesion represents would be constrained and therefore its availability for mitigation may be limited. Once lockdown restrictions were eased then access to that social capital may also be released. We may also speculate that people’s experience was influenced by the narrative of how they should react and process the tragedy around them. There were many communities which strengthened over this time with neighbours helping each other and local benefit groups delivering supplies to those isolating, shielded individuals and the elderly. Not for the first time in a British tragedy, media and politicians made reference to the “Blitz Spirit” and the rhetoric of survival, courage, fortitude and being ’in this together’, using collective actions such as the “Clap for Carers” to further emphasise a message of solidarity. This cultural environment of resilience, may have been a universal protective factor at a national level, facilitating the observed elasticity of mood, moreover at a local level, the removal of traffic from streets, the necessity of restricting contact to only those who you saw on a daily permitted errand or exercise session may have emphasised the importance of living in a cohesive community for mental health, reflected here in the model for recover. Linkov and Trump write about communication as a key factor in resilience ([53], p 109). Effective communication from policy makers and health care systems whilst under stress is critical in encouraging behaviour from the population which does not lead to a breakdown of those mechanisms brought in to reduce risks - in this case, COVID-19 lock down restrictions and guidance on preventing disease transmission. So the outcome observed here is consistent with the relatively good communication during the early pandemic and consequential widespread compliance with pandemic restrictions observed in the first lock down.

The ability of a person to return to normal levels of well being after a negative experience is also considered within the adaptation and coping literatures. The adaptation framework proposes that adverse experience may result in an initial reduction in wellbeing, but over time the person affected can adapt and subjective measures of wellbeing will consequently return to prior levels [54]. Coping theory describes the development of behaviours that aim to reduce stress (although this framework does not presume that any coping mechanism will in fact be successful whereas the concept of adaptation is deemed to be inherently positive) [55]. We acknowledge that adaptation is plausible explanation of the findings, however we prefer an explanation in terms of ’recovery’. Given the real changes in situation as the pandemic progressed, with a lockdown followed by a relaxation which map on to the observed changes, we consider recovery as a more parsimonious theoretical framework for the phenomenon of improving wellbeing scores.

## Strengths and limitations

The sample does not include care home residents and non-response was greater amongst younger people and people from ethnic minority backgrounds. The finding of elasticity cannot therefore be generalised to the whole population. Poor mental well being may well have contributed to the non response and therefore those who were most adversely affected by the pandemic, may have been structurally excluded from the data.

The research uses only those responses submitted online and so this may also exclude certain groups. For many families during the school closures, devices were shared between parents working from home, and children completing online learning. This may have created an additional barrier to completion. For many working from home, even in the absence of competition for access to an appropriate device and with a stable home internet connection, screen fatigue from long hours spent working remotely may have reduced the response rate amongst certain types of workers. Those who do not have an internet connection through choice, or through a lack of means are also excluded here. We have no direct data on these issues but recent work by Schaurer and Weiss [56] did find evidence of selection bias in online survey data collected during the pandemic and so this could have had an impact on our results.

In care homes, many residents live with dementia. To reduce infection risk in this vulnerable population, many in homes and in the community were confined to quarters as quarantine measures took place and there is evidence that this has hastened an irreversible decline in speech, social skills, functional skills and memory [57]. These people are excluded from the analysis and as such the finding that people “bounced back” as restrictions were lifted may not to apply in these contexts.

The remains a stigma around mental health and as such a form of social desirability bias exists within mental health self reporting. This may have impacted on the reliability of the measure used here and there may be some under-reporting within the data. Under-reporting behaviour has been shown to differ between groups and this may therefore have masked signals within the models.

More detailed and targeted data collection is needed to understand the experience of people from ethnic minority backgrounds. The increased non response rate in these data suggest some kind of systematic bias during the COVID-19 data collection. No signal has been found to suggest a differing experience of mental health during the pandemic but this may simply be due to missing responses.

The dataset is rich with additional variables which could have been included, for example the number of children in a household and patterns of domestic work. They are not included in this study however the consideration of the impacts of these and other variables of interest in addition to those considered here is an interesting areas for future research.

We note that recent related work has been carried out by Pierce et al [58]. They also considered five waves of Understanding Society, but used latent class analysis to classify respondents according to GHQ trajectories and then used demographics to predict group membership. They did find some factors that were not significant in our study were predictive of class membership in their analysis. This existing work complements the work we have done here providing a perspective about types of multi point trajectories. However the the key findings of the current paper that the biggest predictor of the initial dip was the baseline score but this was inverted against expectation, (with loneliness being the second biggest predictor) are derived from our focus on specific transitions. Both papers provide useful insights from different perspectives.

## Conclusion

The challenges of social disruption, financial insecurity and changes to our routines resulting from the pandemic might be expected to be long lived and the structures and process of our normal social existence are impacted by contextual risk and pre-existing vulnerabilities [46].

When faced with the unprecedented events of the global pandemic, government sought to implement a risk management strategy, aiming to reduce and mitigate risks from the spread of disease. These measures were restrictive and represented a change to our everyday existence, as well as having widespread economic impact and thus were susceptible to unintended consequences such as a fall in population well being, or a consequent crisis in accessing healthcare. As we continue to experience COVID-19 and its transition to an endemic disease, as a society we are shifting to a resilience model where the systems and individuals are prepared and efforts to control the disease are concentrated on promoting a robust health and social system that reduces the disruption to the economy and to our normal social existence.

There have been clear losses in both the immediate and the long term for many of us. Our social skills, working practices and emotional wellbeing have been challenged by the emergence of this novel disease. These data show however, that in the first wave, these harms have to an extent been mitigated at the population level, and suggest that there has been a return to ’normality’.

This analysis of the COVID-19 survey datasets from the first wave of global pandemic in 2020, show interesting and counterintuitive results. We found no evidence of a social gradient in wellbeing related to the pandemic. In fact, although mental health and wellbeing certainly suffered during the lock down, wellbeing was shown to be highly elastic in this period indicating a national level of resilience which cut across the usually observed health inequalities.

Further research is needed to target those groups who may be excluded from this dataset, but the data would suggest that national efforts to “raise our spirits” may in fact in this context have been useful and effective. This may lend weight to arguments for other nationally led initiatives to improve mood in times of crisis, for example additional national holidays. Critically, over the time period that the COVID-19 datasets were collected a recovery took place. That duration coincided with the first national lock down and the eventual removal of most restrictions for most places in the UK. It would therefore follow that the best policy to improve the nation’s mental well being and to protect vulnerable people from the worst mental illness, is to pursue policies which suppress the pandemic such that the domestic economy can in the widest possible spheres, reopen and people’s pre-pandemic work and social existences can resume.

## Data Availability

The data used in this study were extracted from the Understanding Society survey dataset which may be downloaded from the UK data archive.

## Appendix A

The questions in the General Health Questionnaire module of the Understanding Society participants questionnaire are reproduced here for clarity [7]. Participants are instructed that the questions are about how they have been feeling over the last few weeks.

scghqa [GHQ: concentration]

Have you recently been able to concentrate on whatever you’re doing?

1. Better than usual
2. Same as usual
3. Less than usual
4. Much less than usual

scghqb [GHQ: loss of sleep]

Have you recently lost much sleep over worry?

1. Not at all
2. No more than usual
3. Rather more than usual
4. Much more than usual

scghqc [GHQ: playing a useful role]

Have you recently felt that you were playing a useful part in things?

1. More so than usual
2. Same as usual
3. Less so than usual
4. Much less than usual

scghqd [GHQ: capable of making decisions]

Have you recently felt capable of making decisions about things?

1. More so than usual
2. Same as usual
3. Less so than usual
4. Much less capable

scghqe [GHQ: constantly under strain]

Have you recently felt constantly under strain?

1. Not at all
2. No more than usual
3. Rather more than usual
4. Much more than usual

scghqf [GHQ: problem overcoming difficulties]

Have you recently felt you couldn’t overcome your difficulties?

1. Not at all
2. No more than usual
3. Rather more than usual
4. Much more than usual

scghqg [GHQ: enjoy day-to-day activities]

Have you recently been able to enjoy your normal day-to-day activities?

1. More so than usual
2. Same as usual
3. Less so than usual
4. Much less than usual

scghqh [GHQ: ability to face problems]

Have you recently been able to face up to problems?

1. More so than usual
2. Same as usual
3. Less able than usual
4. Much less able

scghqi [GHQ: unhappy or depressed]

Have you recently been feeling unhappy or depressed?

1. Not at all
2. No more than usual
3. Rather more than usual
4. Much more than usual

scghqj [GHQ: losing confidence]

Have you recently been losing confidence in yourself?

1. Not at all
2. No more than usual
3. Rather more than usual
4. Much more than usual

scghqk [GHQ: believe worthless]

Have you recently been thinking of yourself as a worthless person?

1. Not at all
2. No more than usual
3. Rather more than usual
4. Much more than usual

scghql [GHQ: general happiness]

Have you recently been feeling reasonably happy, all things considered?

1. More so than usual
2. About the same as usual
3. Less so than usual
4. Much less than usual

## Footnotes

1 Non-English cases were excluded from the analysis as deprivation index data is not comparable across the home nations.

2 For more information on the questionnaire see https://www.gl-assessment.co.uk/assessments/products/general-health-questionnaire/

3 There are 8379 valid cases from 6010 unique households. 4553 responses came from respondents where another person in their household also responded to the survey. 3826 respondents were the only respondent in their household—this may be because of non-response or because of them being a sole individual household. We randomly sampled the respondents to create a dataset containing no duplicated households (6010 valid cases) and repeated the modelling. There was no substantive change in the results or findings and so the models are reported here for the full set of valid cases (8379). Household effects are deemed to be negligible in respect of this research although we acknowledge that there may be a household level effect within response rates but this is not captured nor investigated as part of this work.

4 *cc_hhcompc*, *cc_hhcompd* and *cc_hhcompe*.

5 *cc_hhcompa* and *cc_hhcompb*.

6 using the variables *cc_hhearn_period, cc_blhhearn_period, cc_hhearn_amount and cc_blhhearn_amount*. The *_period* variables give information on whether the figures given for household income are weekly, fortnightly, monthly or annual.

7 The variables from Understanding Society used to compile this score were those with the codes: *scopngbhh, nbrcoh3, nbrcoh2 and nbrcoh4*

8 The variables are a mixture of derived, re-coded, calculated, imputed and original variables. For readability all variables have been given plain English names; the relationship between the analysis variables and the ones in the survey dataset can be found in the notes section of Table 1.
